# Associations of Body Mass Index, Motor Performance, and Perceived Athletic Competence with Physical Activity in Normal Weight and Overweight Children

**DOI:** 10.1155/2018/3598321

**Published:** 2018-05-02

**Authors:** Kyle M. Morrison, John Cairney, Joe Eisenmann, Karin Pfeiffer, Dan Gould

**Affiliations:** ^1^Hope College, 222 Fairbanks Ave., Holland, MI, USA; ^2^University of Toronto, 55 Harbord Street, WSB Rm 2044, Toronto, ON, USA; ^3^Volt Athletics, Seattle, WA 98103, USA; ^4^Michigan State University, 27 IM Circle, East Lansing, MI, USA; ^5^Michigan State University, 210 IM Circle, East Lansing, MI, USA

## Abstract

Children who are overweight and obese display lower physical activity levels than normal weight peers. Measures of weight status, perceived motor competence, and motor skill performance have been identified as potential correlates explaining this discrepancy. 1881 children (955 males; 926 females; 9.9 years) were assessed as part of the Physical Health Activity Study Team project. The age, habitual physical activity participation (PAP), body mass index (BMI), socioeconomic status (SES), motor performance (MP), and perceived athletic competence (PAC) of each child included were assessed. Gender-specific linear regression analyses (main effects model) were conducted to identify the percent variance in PAP explained by the following variables: BMI, MP, and PAC. For males, 18.3% of the variance in PAP was explained by BMI, MP, and PAC. PAC explained 17% of the variance, while MP, BMI, and SES only accounted for 0.6%, 0.7%, and 0.5%, respectively. PAC explained 17.5% of PAP variance in females; MP explained 0.8%. BMI, SES, and chronological age were not significant correlates of PAP in girls. An established repertoire of motor skill performance has been seen as a vehicle to PAP in children; however, this study indicates that PAC should not be overlooked in intervention strategies to promote increased PAP.

## 1. Introduction

Childhood obesity is recognized as one of the top public health issues in North America [1, 2]. The prevalence of overweight and obesity among children and adolescents in the United States (U.S.) and Canada is approximately 32%. Although obesity is recognized as a complex multifactorial condition [3], physical inactivity has been identified as a significant factor in the development of obesity [4].

In general, children and adolescents who are overweight and obese have been shown to display lower physical activity levels [5, 6], poorer motor performance (i.e., motor competence, motor ability, and fundamental movement skills) [7–14], and lower perceived motor competence (self-perception of ability to perform motor skills) [11, 13, 14] when compared to their normal weight peers. In turn, motor performance [8, 15–22] and perceived motor or athletic competence [23–25] explain between 5 and 30% of the variance in physical activity. In addition to each correlate individually explaining a portion of the total variance in physical activity, motor performance and perceived motor competence are also synergistically related to physical activity [25, 26]. More specifically, motor performance and perceived motor competence are moderately correlated with each other and also provide an additive effect on the explained variance in physical activity participation. These findings suggest that motor performance abilities and perceived motor competence could be important factors to address when attempting to increase the physical activity levels of children, especially in those who are overweight or obese.

Stodden et al. [27] proposed a model that posits that the risk of becoming overweight and obese is based on the interrelationships amongst physical activity, perceived motor competence, motor competence (motor performance), and health-related fitness. In addition, the model further suggests that if a child is overweight/obese, then he/she may display poor motor performance and low perceived motor competence that could lead to a further decline in physical activity participation. According to Stodden et al. [27], those who possess higher perceived motor competence during early childhood will tend to develop better motor competence. Hence, these children will be more likely to persist when attempting a new task until mastery than a child with inadequate perceived motor competence. As previously noted, children with greater perceived motor competence are also seen to possess greater levels of physical activity. Most notably, in early childhood, motor competence is seen to be greater in children with increased opportunities for participation in physical education, recreational sports, and other physical activities [16]. In addition, the Stodden model [27] suggests that as a child approaches middle childhood and adolescence, poor motor competence negatively impacts perceived motor competence. Older children and/or adolescents that have not established a “strong motor repertoire” may lack the skill(s) and confidence to participate in physical activity. The model suggests that the relationship between perceived and actual motor competence strengthens as a child ages, as does the relationship between motor competence and physical activity. The model ultimately predicts weight status (e.g., healthy weight or unhealthy weight/obesity). However, one specific interest is how the healthy weight outcome is shown to produce a “positive spiral of engagement” in physical activity, which includes children with greater motor competence, higher perceived motor competence, and higher physical activity levels. The model further suggests that unhealthy weight or obesity may be the result of a “perfect storm” of poor perceived motor competence, low motor competence, and low physical activity levels. When a child is of an unhealthy weight or obese, he or she is more likely to disengage in physical activity, which could further exacerbate their weight problem.

As outlined above, several studies have examined the bivariate correlations between physical activity, body mass index (BMI) or weight status, perceived athletic competence, and motor performance; however, there is a need for a multiple regression analysis that examines these variables simultaneously due to their unique synergistic relationship. In particular, due to the interrelationships among motor performance, perceived athletic competence, and physical activity, it is of interest to investigate how the combined influence of motor skills and perceived athletic competence can impact physical activity levels during childhood. The use of multivariate analysis has two major advantages: (1) the relative effect of each variable is assessed, while others are held constant, and (2) the strength of individual variable's ability to predict the outcome (physical activity participation) can be compared, and the strongest predictors might be targeted for intervention.

The purpose of this study is twofold: (1) to examine the relative influence of age, BMI, motor performance, perceived athletic competence, and SES on physical activity participation in children and (2) to examine the interactions among BMI, motor performance, and perceived athletic competence on physical activity participation in children.

## 2. Research Design and Methods

### 2.1. Participants

The Physical Health Activity Study Team (PHAST) project began during the 2004-2005 school year in the Niagara Region of Southern Ontario. Ninety-two schools were contacted to recruit children enrolled in the 4th grade for participation in the study. Seventy-five (83.3%) of the 92 schools granted permission. Informational and consent forms were sent home from school with students. Informed consent was obtained from 2278 (95.8%) of 2378 fourth-grade children enrolled in these schools. All study participants gave verbal assent and had a completed consent form signed by a parent or guardian on file with the primary investigator before data collection began. Training and testing protocols were established during the fall of 2004, and the initial wave of data collection occurred in the spring (April and May) of 2005.

Data collected on 2190 children (1104 males; 1086 females) in the 4th grade (aged 8 to 11 years old) during the spring of 2005 were included in this analysis. Of the 2190 participants assessed in the PHAST spring 2005 cohort, 1881 children (955 males; 926 females) had values recorded for age, height, weight, motor performance testing, perceived athletic competence, physical activity, and socioeconomic status. Participants were included in the current analyses only if they had complete data for all of the abovementioned variables. There were 213 participants with missing motor performance values, which was the largest missing variable amongst the measures. Comparisons between participants with complete and missing data are shown in Table 1. There were no statistically significant differences in age, body size, physical activity, or household income; however, perceived athletic competence score (18.5 ± 4.1 versus 17.9 ± 4.1; *p*=0.024) and the motor performance percentile (66.9 ±30.0 versus 52.5 ± 33.6; *p* < 0.001) were significantly higher in those with complete data compared to those with incomplete data.

### 2.2. Measurement of the Outcome Variable: Habitual Physical Activity

The Physical Activity Participation Questionnaire (PAQ) is a 63-item self-reported questionnaire that seeks to assess the participation levels of children in free-time play, intramural school sports, community and club sports teams, and all other organized physical activities (PA) recalled from the previous year. Higher PAQ scores indicate a greater number of “activity units.” The total score ranges from 0 to 45 with a free-play index from 0 to 16 and an organized activities index from 0 to 29. Free play is assessed by recalling typical activity choices and organized activities catalog participation in organized athletic and competitive activities over the previous year.

Two-week test-retest reliability of the PAQ among children in primary grades four through six has been found to be *r*=0.81 [28]. The PAQ has also shown moderate correlation (*r*=0.62) to teacher evaluation of activity participation; however, it has not been validated against an objective measure of physical activity (e.g., accelerometer). Furthermore, the instrument has good construct validity with expected differences between genders and between individuals living in different geographic locations (urban versus rural) [28]. For this study, the PAQ was administered in a classroom setting with a brief description of instructions by research assistants, who were available to answer questions and provide assistance when needed.

### 2.3. Assessment of Physical Activity Correlates

#### 2.3.1. Age

Chronological age (years) was calculated as the decimal age (observation date minus birthdate).

#### 2.3.2. Anthropometry

Height and weight were measured according to standard procedures. Height was measured to the nearest 0.2 cm using a portable stadiometer (SECA, Hamburg, Germany) without the child wearing shoes. Children stood vertically erect with heels together, eyes forward, shoulders relaxed, and arms at their sides. Weight was measured to the nearest 0.1 kg using a calibrated electronic scale (Tanita, Tokyo, Japan). Children wore athletic shorts and T-shirt, which were standard for their physical education classes. These testing sessions occurred in a private testing area at prescheduled times.

The BMI was calculated using the following equation: body weight in kg/height in m^2^. Age- and gender-specific BMI cutoff points [29] were used to determine classification of BMI percentiles into one of the two groups: normal weight (NW) <85th percentile and overweight/obese (OW/OB) ≥85th percentile. Participants with weight status classified as underweight (<5th age- and gender-specific percentile) were included in the normal weight group. This decision was made because there were only 12 males and 22 females classified as underweight, and more importantly, there were no significant differences (other than body mass index) when comparing gender-specific mean values of all variables by weight status (<5th percentile versus ≥5th percentile).

#### 2.3.3. Motor Performance

Motor performance was assessed using the Bruininks-Oseretsky Test of Motor Proficiency-Short Form (BOTMP-SF) [30]. The BOTMP-SF is a well-known and well-accepted [31, 32] product-oriented test used to assess fine and gross motor skills for children between the ages of 4 and 21 years. The short form of the test has shown strong validity (*r*=0.90–0.91) when compared against the long form in children aged 8 to 14 [30]. The BOTMP-SF consists of 14 items from all 8 subtests of the complete form which include standing on the preferred leg on a balance beam, walking heel/toe on a balance beam, tapping feet while making circle with fingers, jumping up and clapping hands, standing broad jump, catching a tossed ball with both hands, throwing a ball at a target with the preferred hand, running speed and agility (shuttle run), response speed, drawing a line through a straight path with the preferred hand, copying a circle with the preferred hand, copying overlapping pencils with the preferred hand, sorting shape cards with the preferred hand, and making dots in circles with the preferred hand. The raw scores from each of the 8 subtests are converted to a scale score which then can be used to establish a percentile rank (i.e., the 77th percentile) or a standard score ranging from 24 to 75 for each subtest by the age group in 6-month intervals from 4.6 to 14.5 years of age. The percentile rank of each participant was used as the measure of motor performance for the analyses within this study.

Prior to conducting motor performance evaluations in the PHAST study, a subset of the research assistants were trained by a motor developmentalist experienced in conducting the BOTMP-SF protocol. The motor testing of PHAST study participants was completed with one child at a time. However, due to conducting assessments in 75 different schools, the testing environment varied. The setting that was selected at each school was chosen to allow open space, minimize distraction, and maximize privacy.

In an attempt to validate the BOTMP testing in this study, 24 children were reassessed by a pediatric occupational therapist, and the testing procedures and findings were supported by the clinician. Two years after initial assessment, 77 children were also selected from a randomly selected subset of schools in the PHAST study. These participants' results were retested by different examiners blinded to the original BOTMP-SF results; the correlation between the two sets of scores was 0.70 (*p* < 0.001). This demonstrated that the relative percentile rank of children tracked moderately well.

#### 2.3.4. Perceived Athletic Competence

The Self-Perception Profile for Children (SPPC) [33] was designed to evaluate self-perception of children in multiple dimensions. The assessment contains five six-item subscales that evaluate perceived competence in the following dimensions: athletic, scholastic, and social competence, as well as physical appearance and behavioral conduct. The scoring system utilizes a 4-point scale in which the participant must first decide which of two statements best describes them and then indicate whether the statement is “sort of true” or “really true” for them. Each item can be scored from 1 (low self-perception) to 4 (high self-perception). Both the total subscale and average subscale scores can be reported. The Self-Perception Profile for Children states that the “athletic competence items primarily refer to one's ability to do well at sports, including outdoor games, demonstrating one's athletic prowess.” The perceived athletic competence subscale relates most closely to performance of motor skills and their application to sports participation. Therefore, since a true measure of perceived motor competence was not utilized in the PHAST study, the perceived athletic competence (PAC) total subscale score was used in this analysis. It possesses test-retest reliabilities that range from *r*=0.76  to  0.91 depending on the sample.

#### 2.3.5. Socioeconomic Status

In this study, neighborhood income was used as a marker of SES. SES is often measured using the level of parental education, parental occupation, or household income. We chose to focus on income because in this context, we are specifically interested in the ability to pay for access and participation in organized sports and recreational programs. Certainly, previous research has shown that participation in organized sports and physical activity is lower in low-income neighborhoods as compared to high-income neighborhoods [34] Moreover, White and McTeer [35] found household income to be the strongest predictor of children's participation in organized sports.

In this study, instead of parent-reported income or occupation, the reported residential postal code of each child was recorded. The postal codes were then used to generate proxy estimates for household income based on census information. Postal codes were geocoded in ArcMap using the North America Geocode Service from Esri. Mean household income data were obtained from the 2006 Census of Canada according to the dissemination area (one or more blocks with a population between 400 and 700 people) associated with the postal code reported. Neighborhood income has been shown as a valid proxy for household income in population studies, especially in relation to health-related outcomes [36].

### 2.4. Statistical Analysis

Descriptive statistics were calculated for all variables. To examine the relationship amongst variables, a Pearson correlation matrix was created for each gender. Further, a forced regression analysis (main effects model) was conducted to identify the percent variance in habitual physical activity participation explained by the following variables: BMI, motor performance, and perceived athletic competence. To control for the effect of socioeconomic status and decimal age, these variables were placed in separate blocks of independent variables within the regression model. Based on % variance explained in bivariate relationships discussed from previous literature, it was hypothesized that each of the potential correlates would explain greater than 5% of the variance in physical activity, with the complete model explaining 20% of the variance. Variance inflation factors (VIFs) were calculated to assess for multicollinearity between the independent variables in the final model. Variance inflation factors between the independent variables less than 10.0 are considered to be free of multicollinearity.

A three-way interaction term (BMI × motor performance × perceived athletic competence) was computed as were all lower-level two-way interaction terms. The main effects and two-way and three-way variables were force entered into a multiple regression equation (interactions model) to identify the percent variance in physical activity. To control for the variance of socioeconomic status and chronological age, these variables were placed in a separate block of independent variables within the regression model. Variance inflation factors were calculated to assess for multicollinearity between the independent variables (main effects, 2-way interaction terms, and a 3-way term) in the final model.

The likelihood of multicollinearity being present in an interactions model is very high due to the fact that each independent variable is entered into the regression analysis multiple times (main effects, 2-way interactions, and 3-way interaction). Therefore, in an attempt to reduce the likelihood of multicollinearity being present between the variables, the technique of centering was implemented. Centering of the independent variables included in the 3-way interaction was conducted by subtracting each subject's score from the gender-specific mean value of the variable. After this process, the interactions model was rerun and VIFs were checked again.

All analyses were conducted using the Statistical Package for the Social Sciences (SPSS) Version 19.0. Significant differences will be determined by a *p* value less than 0.05.

## 3. Results

Descriptive statistics for the total sample and by gender are presented in Table 2. Although age, height, weight, and BMI were similar between genders, boys had a significantly higher mean BMI percentile (63.6 ± 27.4) when compared to girls (60.6 ± 29.6). Less than 2% of all participants were classified as underweight, 68% were normal weight (NW), 15.8% were overweight, and 14.4% were obese, and these percentages were similar between genders. There were no significant differences in PA participation between genders. Girls had significantly lower PAC (17.8 ± 4.3 versus 19.1 ± 3.9; *p* ≤ 0.001) and MP (62.6 ± 30.4 versus 71.2 ± 29.00; *p* < 0.001) than boys.

Pearson correlation coefficients amongst variables are reported in Table 3 for boys and girls. In boys, PA participation was significantly (*p* < 0.001) correlated with PAC, MP, and SES but not with BMI. Correlation coefficients were low between PA participation and both MP (0.191) and SES (0.116), but there was a moderate relationship between PAC and PA (0.413). Other significant (*p* < 0.001) correlations were shown between PAC and MP (0.267) and BMI and MP (−0.316). In girls, PA participation was significantly correlated with PAC, MP, BMI, and SES. Correlation coefficients were low between PA and MP (0.185; *p* < 0.001), BMI (−0.071; *p* < 0.05), and SES (0.050; *p* < 0.01), but PAC was moderately correlated with PA (0.420; *p* < 0.001). Other significant but weak correlations were found between PAC and MP (0.224; *p* < 0.001), as well as between BMI and motor performance (−0.237; *p* < 0.001).

Results of the linear regression analysis with forced entry (main effects model) for physical activity participation in boys and girls are presented in Table 4. For boys, 18.3% of the variance in habitual physical activity participation was explained by BMI, MP, and PAC. When socioeconomic status was included in the model, the total variance explained was 18.8%. When using a stepwise approach, PAC independently explained 17% of the variance, while MP, BMI, and SES only accounted for 0.6%, 0.7%, and 0.5% of the total variance, respectively. Chronological age was not a significant predictor of physical activity participation. Similar to boys, PAC was also the most robust predictor of physical activity participation in girls explaining 17.5% of the variance. MP contributed an additional 0.8%. BMI, SES, and chronological age were not statistically significant correlates of physical activity participation in girls. All VIFs in the main effects models ranged from 1 to 1.2, demonstrating that multicollinearity was not present between correlates.

All main effects and two-way interaction and three-way interaction terms were examined in the forced multiple regression analysis (interactions model) for physical activity participation in boys and girls. When the main effects and interaction terms were uncentered, there were no significant findings and VIFs ranged from 50 to 781, which indicates that multicollinearity was present. In an attempt to reduce multicollinearity and reduce VIFs, all main effects were centered. The two-way and three-way interaction terms were recalculated using centered main effects, and the model was rerun. For boys, the percent variance in habitual physical activity participation explained by the 3-way interactions model was 18.3%. However, the only predictors with significant beta values were the centered values for BMI, MP, and PAC, which was what the main effects model demonstrated. For girls, the centered multiple regression model explained 18.1%, but the only predictors with significant beta values were the centered values for MP and PAC. None of the 2-way interactions terms or the 3-way interaction were statistically significant.

## 4. Discussion

This study examined the relative influence of age, BMI, MP, PAC, and SES and their interactions on physical activity participation in a large sample of children. The main finding was that PAC explained approximately 17% of the total variance of participation in physical activity. The interactions models produced no significant results.

The prevalence of overweight (15.8%) for boys and girls was slightly lower than that reported in a nationally representative sample of 5- to 11-year-old Canadian boys (19.8%) and girls (19.6%) [37]. Furthermore, significant differences exist in obesity prevalence between boys (19.5%) and girls (6.3%) in Canadian national data [37]. However, these were not present in the current study as both genders had an obesity prevalence of 14.5%. The MP centiles for boys (71st) and girls (63rd) in the current study are both classified at the upper end of the “average” classification (18th to 83rd centiles) for age-specific normative data [38]. The differences in MP between girls and boys observed here confirm the well-known finding that boys possess greater MP abilities than girls [39]. The PAC values in the current study are similar to those found in similarly aged children [33, 40–42]. Significant differences in PAC between girls and boys observed here also confirm previous reports [25, 33, 41]. The PAQ scores of children (15.4) in this sample were lower than the range of values (17.5–30.0) that was presented for children in the 4th through 6th grades in the instrument reference data [28]. The PAQ scores of girls (15.5) in the current study were similar to scores of a sample of adolescent girls (15.2) from the same geographic region; however, boys (15.5) had lower scores than the adolescent boys (24.4) in the comparison study [43]. The difference in scores in boys may be due to an increase in opportunities for school-sponsored sports teams upon entering middle and high school. Finally, no differences in physical activity participation were observed between genders in the current study. However, significant differences in physical activity have been reported between boys and girls previously using the physical activity participation questionnaire [43], as well as using different assessments of physical activity [5, 6].

Findings from previous studies suggest that the relationship between MP and PA is in general modest, but the explained variance ranges from 1 to 30% of the variance in physical activity [8, 15–22]. Despite being a significant correlate of physical activity participation (*r*=0.19), MP explained a small proportion of the total variance in physical activity (<1%) in the regression model of this study. Similarly, Barnett et al. [44] found that physical activity assessed by the questionnaire and locomotor skill performance were weakly correlated (*r*=0.14) in adolescents (16.2 years), explaining only 2% of the variance in physical activity. However, object-control skill performance (skill requiring control of an object with part of the body or an implement) (*r*=0.35; *p* < 0.01) explained over 12% of the variance in physical activity. This difference in variance may be due to the complexity and ballistic, sport-specific nature of object-control skills compared to the rudimentary nature of locomotor skills.

In general, previous studies suggest that the relationship between MP and PA is modest but can vary due to the population assessed as evident from the range of variance [8, 15–22]. Stronger correlations have been observed in younger children [21] compared to adolescents [22] and in males compared to females [20]. Finally, the utilization of the product-oriented MP test and a child-reported questionnaire to assess PA may have weakened the relationship in this population as the most robust relationships demonstrated in previous studies [20, 21] have been those that objectively measured PA (i.e., accelerometers) and/or used process-oriented MP assessments to determine the developmental level.

Besides MP, PAC, specifically addressing the predictive ability of physical self-perception, has also been found to explain 7 to 29% of the variance in physical activity levels of children [23–25]. In the current study, PAC was the most robust correlate of PA in both boys and girls, with the explained variance (∼17%) falling within the range of previous investigations (i.e., 7–29%). Crocker et al. [24] found that self-perceptions of physical conditioning and sport skills had moderately strong correlations (*r*=0.46  to  0.48) with 7-day PA recall scores in 10- to 14-year-old Canadian youth. These findings are in close agreement with the current study utilizing similar methodologies. In a longitudinal study, Davison et al. [25] observed a slightly weaker correlation (*r*=0.27) between perceived motor competence of girls at 9 years of age and physical activity at 11 years of age. Barnett et al. [44] assessed physical activity participation in relation to perceived sports competence in 215 adolescents and found a moderate correlation (*r*=0.31) with MVPA. The findings from these previous investigations and the current study confirm that PAC is a moderate predictor of physical activity; however, the strength of the relationship may vary by age. When children experience successful performance of fundamental motor skills, they may display improvements in PAC [45]. Furthermore, the mastery of fundamental motor skills may also increase motivation to be physically active due to improvement of self-esteem and enjoyment in participation [9].

The MP abilities [8–10, 46] and PAC [11, 47] of children who are overweight and obese are generally lower in comparison to their normal weight peers. The current study also found an inverse relationship between BMI and both MP and PAC. However, the relationship was stronger for MP (*r*=−0.316 (boys), *p* < 0.001; *r*=−0.237 (girls), *p* < 0.001) than PAC (*r*=−0.044 (boys), NS; *r*=−0.071 (girls), *p* < 0.05).

The bivariate relationships discussed above between MP, perceived motor competence, BMI, and physical activity have been summarized by Stodden et al. [27]. Furthermore, the synergistic relationship between MP and perceived motor competence [25, 26] has also been found in children. Therefore, it was proposed that the three-way interaction between PAC, MP, and BMI may explain a considerable amount of the physical activity participation of children. However, neither the 3-way interaction term nor any lower-level 2-way interactions were significant predictors of physical activity participation as hypothesized. Similarly, Morgan et al. [20] assessed the amount of variance that chronological age, BMI *z*-score, motor competence, and PAC could explain in objectively measured PA in youth who are obese. Object-control proficiency explained 25% and 10% of the variance, respectively, of accelerometer counts per minute and % of observed time spent in VPA for boys. For girls, age was the only significant predictor of MPA and VPA, explaining 38% and 15%, respectively. BMI *z*-score and PAC were not identified as statistically significant correlates of physical activity. All two-way interactions between age, MP variables, and BMI *z*-score were assessed as covariates of MPA, VPA, and CPM, but none were found to be significant. The results of the Morgan study [20] provide support that chronological age and MP assessed with a process-oriented instrument could be significant correlates of objective-measured physical activity during childhood. However, the subjects in this study were enrolled in an obesity intervention and thus may not be representative of the children who are obese or the general population. The current study undertook a similar investigation using a representative sample of youth across the BMI spectrum. The results indicated that PAC had a much greater ability to predict physical activity participation in children than actual MP, SES, and BMI. Therefore, targeting improvements in PAC may be a worthwhile objective in physical activity interventions for OW/OB children who have reached an age where limited plasticity in MP exists [48, 49] such as that in the current study.

Previous intervention programs specifically targeting children and adolescents who are overweight and obese have shown improvements in both motor performance and perceived athletic competence outcomes [47, 50]. In a 10-week intervention [50], significant pre- to postprogram improvements in gross motor quotient and perceived athletic competence were found in thirteen children who are overweight and obese (10.4 years ± 1.2 years) and were still apparent at 9-month follow-up. However, despite the improvements in motor performance and athletic perceived competence, the program did not demonstrate the ability to reduce BMI or improve PA participation. In fact, there was a significant decline in minutes of MVPA from baseline to postprogram and again from postprogram to follow-up. However, an outpatient clinical program in Italy assessed forty-one children (9.2 ± 1.2 years) before and after an 8-month, 80-session physical training program [47]. Significant increases were observed in PA, MP, and PAC, while BMI decreased. Findings from these intervention programs indicate that PAC and MP abilities can be improved in children who are overweight and obese. The greatest improvements were typically associated with programs of longer duration, but regardless of the program length, improvements in MP abilities and PAC deteriorated by long-term follow-up. Further investigation into the long-term impact these intervention programs have on PA participation and associated correlates (MP, PAC, and weight status/body composition) is warranted.

The current study had several limitations. There were multiple staff conducting the anthropometric assessments, and interrater reliability was not determined. However, there was a consistent training and testing protocol. Several participants in the original PHAST study had incomplete data, and there were significant mean differences in MP and PAC between those with and without complete data, which could have biased the sample in this analysis. Finally, there are a variety of instruments (objective versus subjective measures) available to assess physical activity participation of children. Instruments used to define the associated correlates (body composition, MP, PAC, and perceived motor competence) of physical activity also vary in sophistication and utility. In the current study, physical activity participation was evaluated using a validated, self-reported measure in the current study. Despite this instrument showing good test-retest reliability, it did not possess the strength of correlation with the correlates of physical activity analyzed in this study that previous studies using objective measures have shown. Finally, motor performance was assessed using a product-oriented test which may not have sufficiently assessed the developmental motor proficiency of fundamental motor skills that a process-oriented instrument typically does.

Despite these shortcomings, there were also several strengths of this study. This study was the first to assess the interactions between BMI, PAC, and MP in a large representative sample of children, which allows for improved generalizability to the general population. Subjects in this study were older than the development age of 7.9 years at which motor development abilities have been suggested to begin stabilizing and approach the mature state [48, 49, 51]. Therefore, the impact of developmental age on MP may have been reduced.

Motor performance abilities have been previously identified as an important target when attempting to increase physical activity levels of children and adolescents [50, 52, 53]; however, the perception of one's ability to perform motor skills should not be overlooked. The synergistic relationship between perception of MP and actual MP has been highlighted and should be considered when designing interventions. This study showed that PAC is an important predictor of physical activity in all children, and specific attention should be given to improving self-perception of motor abilities in children who are overweight and obese. When children have a sense of confidence and self-belief in their ability to engage in sports skills, they will be more likely to participate in physical activity.

Although the results of this study indicate that only PAC has an impact on the physical activity levels of children, a strong repertoire of MP abilities has also been documented to promote physical activity in children who are obese [20]. Furthermore, poor motor ability and low perception of physical ability may impact a child's psychological outlook and both current and future participation in leisure physical activity and recreational sports. The differences in MP and PAC across weight status may be one specific reason for the steep decline in physical activity frequently observed during adolescence [54]. Longitudinal studies may be necessary to identify how these two correlates impact physical activity participation by weight status across the childhood and adolescence.

## Figures and Tables

**Table 1:** Descriptive characteristics of participants with complete and incomplete data.

Variable	Total		Complete data		Incomplete data	
	*n*	Mean (SD)	*n*	Mean (SD)	*n*	Mean (SD)
Decimal age (years)	2190	9.9 (0.4)	1881	9.9 (0.4)	309	9.9 (0.4)
Height (cm)	2189	139.3 (6.5)	1881	139.4 (6.5)	308	139.1 (6.7)
Weight (kg)	2190	36.4 (9.0)	1881	36.4 (8.9)	309	36.4 (9.2)
Body mass index (kg/m^2^)	2189	18.6 (3.5)	1881	18.6 (3.5)	308	18.6 (3.6)
Body mass index percentile	2189	62.0 (28.7)	1881	62.1 (28.6)	308	61.2 (29.8)
Perceived athletic competence	2176	18.4 (4.1)	1881	18.5 (4.1)^∗^	295	17.9 (4.1)
Motor performance percentile	1977	66.2 (30.3)	1881	66.9 (3.0)^∗^	96	52.5 (33.6)
Average area household income ($)	2074	70547.45 (21566.97)	1881	70643.13 (21565.57)	193	69614.87 (21614.41)
Physical activity participation score	2177	15.4 (6.7)	1881	15.4 (6.7)	296	15.2 (6.9)

^∗^ *p* < 0.05.

**Table 2:** Descriptive characteristics of the total sample and boys and girls in the analytic sample.

Variable	Total		Boys		Girls	
	*n*	Mean (SD)	*n*	Mean (SD)	*n*	Mean (SD)
Decimal age (years)	1881	9.9 (0.4)	955	9.9 (0.4)	926	9.9 (0.3)
Height (cm)	1881	139.4 (6.5)	955	139.5 (6.2)	926	139.3 (6.9)
Weight (kg)	1881	36.4 (8.9)	955	36.3 (8.5)	926	36.5 (9.3)
Body mass index (kg/m^2^)	1881	18.6 (3.5)	955	18.5 (3.4)	926	18.6 (3.6)
Body mass index percentile	1881	62.1 (28.6)	955	63.6 (27.4)^∗^	926	60.6 (29.6)
*Underweight*	34	1.8%	12	1.3%	22	2.4%
*Normal weight*	1279	68.0%	654	68.5%	625	67.5%
*Overweight*	297	15.8%	151	15.8%	146	15.8%
*Obese*	271	14.4%	138	14.5%	133	14.4%
Perceived athletic competence	1881	18.5 (4.2)	955	19.1 (3.9)^∗^	926	17.8 (4.3)
Motor performance percentile	1881	66.9 (30.0)	955	71.2 (29.0)^∗^	926	62.6 (30.4)
Average area household income ($)	1881	70643.13 (21565.57)	955	70645.23 (21844.44)	926	70640.97 (21285.94)
Physical activity participation score	1881	15.4 (6.7)	955	15.5 (6.9)	926	15.2 (6.5)

^∗^ *p* < 0.05.

**Table 3:** Bivariate correlation coefficients between physical activity and potential correlates in boys (*n*=955) and girls (*n*=926).

	Perceived athletic competence		BOTMP percentile		BMI		Average area household income	
	Boys	Girls	Boys	Girls	Boys	Girls	Boys	Girls
PA Participation Questionnaire Score	0.413^∗∗∗^	0.420^∗∗∗^	0.191^∗∗∗^	0.185^∗∗∗^	0.038	−0.071^∗^	0.116^∗∗∗^	0.050^∗∗^
Perceived athletic competence			0.267^∗∗∗^	0.224^∗∗∗^	−0.044	−0.071^∗^	0.084^∗∗^	0.0911
Motor performance percentile					−0.316^∗∗∗^	−0.237^∗∗∗^	0.093^∗∗^	0.056^∗^
Body mass index							−0.055^∗^	−0.042
Average area household income								

^∗^ *p* < 0.05; ^∗∗^*p* < 0.01; ^∗∗∗^*p* < 0.001.

**Table 4:** Significant main effects for correlates of physical activity participation of boys (*n*=955) and girls (*n*=926).

Boys	Unstandardized *β*	*p* value	Variance explained
*Model A*			Model-adjusted *R*^2^ = 0.183
Perceived athletic competence	0.688 (0.054)	0.000	Adjusted *R*^2^ = 0.170
Motor performance	0.028 (0.008)	0.000	Adjusted *R*^2^ = 0.006
Body mass index	0.183 (0.062)	0.003	Adjusted *R*^2^ = 0.007
*Model B*			Model-adjusted *R*^2^ = 0.188
Perceived athletic competence	0.679 (0.054)	0.000	Adjusted *R*^2^ = 0.170
Motor performance	0.027 (0.008)	0.000	Adjusted *R*^2^ = 0.006
Body mass index	0.188 (0.062)	0.002	Adjusted *R*^2^ = 0.007
Socioeconomic status	0.0000247 (0.000)	0.008	Adjusted *R*^2^ = 0.005
*Girls*			Model-adjusted *R*^2^ = 0.183
Perceived athletic competence	0.595 (0.046)	0.000	Adjusted *R*^2^ = 0.175
Motor performance	0.021 (0.007)	0.002	Adjusted *R*^2^ = 0.008

## Data Availability

This study was carried out using data from the Physical Health Activity Study Team (PHAST) project as part of the doctoral dissertation of Dr. M. Morrison; the link for the electronically published version is used with permission: https://d.lib.msu.edu/etd/3563. Data inquiries can be sent to Dr. John Cairney at john.cairney@utoronto.ca or Dr. John Hay at jhay@brocku.ca.
